# CANOES: detecting rare copy number variants from whole exome sequencing data

**DOI:** 10.1093/nar/gku345

**Published:** 2014-04-25

**Authors:** Daniel Backenroth, Jason Homsy, Laura R. Murillo, Joe Glessner, Edwin Lin, Martina Brueckner, Richard Lifton, Elizabeth Goldmuntz, Wendy K. Chung, Yufeng Shen

**Affiliations:** 1 Departments of Systems Biology and Biomedical Informatics, Columbia University Medical Center, New York, NY 10032, USA; 2 JP Sulzberger Columbia Genome Center, Columbia University Medical Center, New York, NY 10032, USA; 3 Cardiovascular Research Center, Massachusetts General Hospital, Boston, MA 02114, USA; 4 Department of Genetics, Harvard Medical School, Boston, MA 02115, USA; 5 Departments of Pediatrics and Genetics and Genomic Sciences, Icahn School of Medicine at Mount Sinai, New York, NY 10029, USA; 6 Center for Applied Genomics, Children's Hospital of Philadelphia, Philadelphia, PA 19104, USA; 7 Departments of Pediatrics and Medicine, Columbia University Medical Center, New York, NY 10032, USA; 8 Department of Genetics, Yale University School of Medicine, New Haven, CT 06510, USA; 9 Howard Hughes Medical Institute, Yale University, New Haven, CT 06510, USA; 10 Department of Pediatrics, Perelman School of Medicine at the University of Pennsylvania, Philadelphia, PA 19104, USA

## Abstract

We present CANOES, an algorithm for the detection of rare copy number variants from exome sequencing data. CANOES models read counts using a negative binomial distribution and estimates variance of the read counts using a regression-based approach based on selected reference samples in a given dataset. We test CANOES on a family-based exome sequencing dataset, and show that its sensitivity and specificity is comparable to that of XHMM. Moreover, the method is complementary to Gaussian approximation-based methods (e.g. XHMM or CoNIFER). When CANOES is used in combination with these methods, it will be possible to produce high accuracy calls, as demonstrated by a much reduced and more realistic *de novo* rate in results from trio data.

## INTRODUCTION

Copy number variants (CNVs) play a key role in human disease. Rare CNVs may account for ∼15% of cases of pediatric neurodevelopmental disease (1). A recent study found that severe obesity is often associated with a significant burden of large rare CNVs (2). Although both rare and common CNVs are thought to carry substantial risk for disease, much recent activity has focused on the role played in disease by rare CNVs, given the smaller cohort sizes required to attain statistical significance for identifying highly penetrant risk-associated rare CNVs (3–7).

To date, microarray-based approaches have been most commonly used for the genome-wide detection of rare CNVs (3,8). With the advent of large-scale whole exome sequencing studies, however, several methods have become available to use exome sequencing data to detect rare CNVs (9,10). In exome sequencing, the DNA in targeted regions (targets) consisting of the exons and other selected genomic regions is captured and sequenced, producing sequence data for non-contiguous regions spread across the genome (11). These sequence data can be used to detect CNVs, because the depth of sequence coverage at any target is generally correlated with the copy number at that target. CNV detection is complicated by target and sample-specific biases caused by guanine-cytosine content (GC content), sequencing conditions and other factors, which lead to a high level of background variability in sequence coverage even in the absence of CNVs. However, different samples, particularly if they are sequenced under similar conditions, will generally have highly correlated sequence depth at the same target. This correlation can be used to construct a model, for any particular sample, of what the sequence depth at any particular target should be in the absence of a CNV (12).

The existing methods that focus on the detection of rare CNVs from exome sequencing data generally require sequence depth data from at least 50 samples and use principal components analysis (PCA) to normalize the data and remove the principal modes in which sequence depth varies among samples and targets (CoNIFER (9)). These methods have been reported to have good performance. For example, a recent study showed that one such method, XHMM , was able to detect 79% of rare CNVs called with high confidence using microarray data (10). These methods rely on a Gaussian approximation of the distribution of sequence depth. This is not an accurate model at typical depth of coverage, which at some targets may be quite low (13). The Gaussian approximation may therefore lead to loss of power of detection, particularly for small deletions (Supplementary Table S1). To address this deficiency, we provide an algorithm, CANOES (CNVs with an Arbitrary Number Of Exome Samples), that models sequence coverage using the negative binomial distribution, which has been found to be a good model for overdispersed sequence depth data (13–16). We demonstrate the method by applying it to a family-based exome sequencing dataset and show how it compares to XHMM using CNVs called by PennCNV from genotyping microarrays as the comparator (17).

## MATERIALS AND METHODS

### Read count model

**Figure 1.**
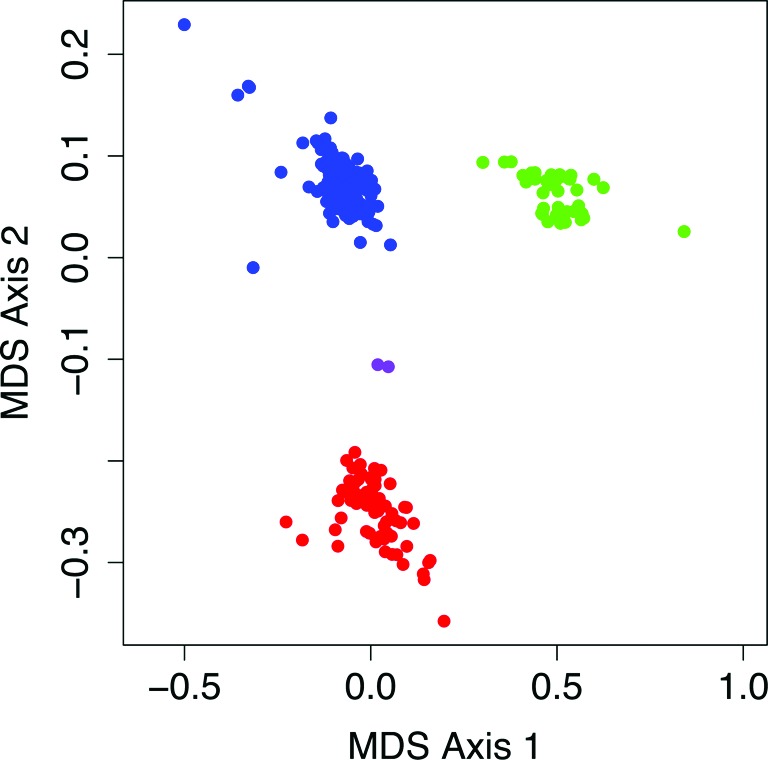
Batch effects. The scatter plot shows the results of a 2D scaling of the inverse of the covariance matrix of the read count data vectors $K_{.j}$$K_{.j}$ using R's *cmdscale* function, for the dataset described in ‘Results’ section. One sample relatively uncorrelated with all the other samples (highest pairwise correlation 0.82) has been omitted from the plot. Within the three major clusters, the pairwise correlation between samples often exceeds 0.99. The colors are used to distinguish between the various clusters in relevant plots in the Supplementary materials.

Our workflow is conceptually illustrated in the flowchart in Supplementary Figure S1. We assume that the number of reads *K_ij_* in sample *j* that overlap with capture target *i* can be modeled by a negative binomial (NB) distribution, $K_{ij} \sim {\rm NB}(\mu _{ij}, \sigma _{ij}^2)$$K_{ij} \sim {\rm NB}(\mu _{ij}, \sigma _{ij}^2)$, which has two parameters, the mean *μ_ij_* and the variance $\sigma _{ij}^2$$\sigma _{ij}^2$. If the read count data of all the available reference samples were equally well correlated with the read count data of sample *j*, these parameters could be estimated by calculating the sample mean and variance of the read count of all those samples at target *i*. However, different sets of samples are subject to different systematic read count biases. Figure 1 and Supplementary Figure S2 illustrate that, for the dataset described further in ‘Results’ section, there are three batches of samples that each have similar read count biases, along with several outlier samples.

To account for these factors, we first normalize the read count data for each reference sample *k* to have the same aggregate read count (over all targets) as sample *j*. *μ_ij_* and the variance $\sigma _{ij}^2$$\sigma _{ij}^2$ are then given by a weighted mean and variance (calculated using the *Hmisc* package in R) of the normalized read count data for the reference samples, where reference sample *k* is assigned a weight *w_k_*. We aim to weight reference samples more highly to the extent they share the systematic read count biases of sample *j*. To accomplish this, we regress the read count data of sample *j* against the read count data of the reference samples using non-negative least squares regression (using the *nnls* package in R), which constrains the coefficients to be positive and minimizes multicollinearity. Because we have normalized the read count data so that all reference samples have the same aggregate read count as sample *j*, the coefficients also sum to 1. We then use these coefficients as the weights *w_k_*. (For a different approach to the same problem, which does not use weighting, see (13).)

When the effective number (taking into account the weighting discussed above) of reference samples used to estimate $\sigma _{ij}^2$$\sigma _{ij}^2$ is low, the prediction of $\sigma _{ij}^2$$\sigma _{ij}^2$ may be inaccurate and sometimes may be even lower than *μ_ij_*, implying variance of read count less than Poisson, in which case the variance would equal the mean. We therefore set a floor on the estimate of $\sigma _{ij}^2$$\sigma _{ij}^2$ equal to the higher of the mean read count at target *i* (*μ_ij_*) and $s_{ij}^2$$s_{ij}^2$. $s_{ij}^2$$s_{ij}^2$ is a prediction of $\sigma _{ij}^2$$\sigma _{ij}^2$ based on the observed relationship between the sample variance and two covariates, the sample mean and the GC content. We use this floor to improve the prediction of $s_{ij}^2$$s_{ij}^2$ by incorporating information from all the targets with similar mean sequence depth and GC content to target *i* (14).

**Figure 2.**
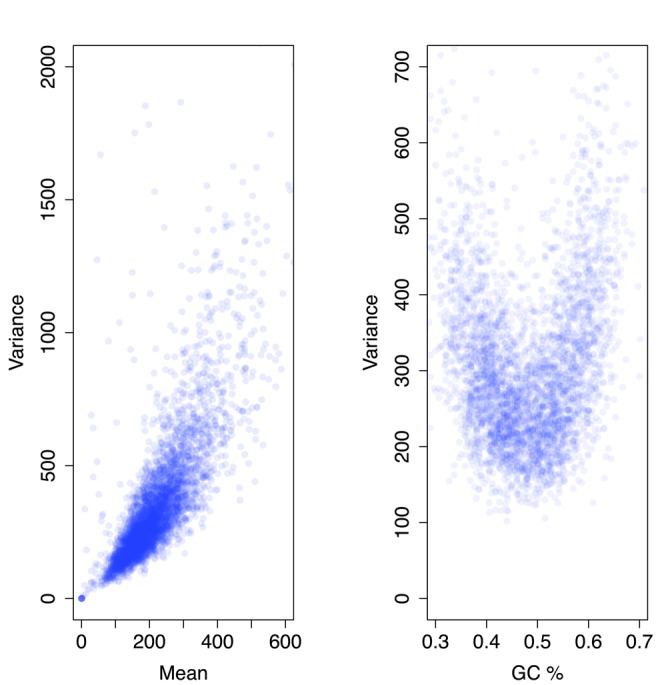
Relationship of variance to mean read count and GC content. The scatter plot on the left shows that the variance of the read count at a target across reference samples increases curvilinearly with the mean read count at that target. For this plot, only targets with median GC content were included. The scatter plot on the right shows that the variance of the read count is higher at low and at high GC content.

The variance of read count of targets in the reference samples is observed to increase in a curvilinear fashion with the mean read count and to be higher at both high and low GC content (see Figure 2). To establish a floor on the estimate of $\sigma _{ij}^2$$\sigma _{ij}^2$ at a target *i* of mean read count *μ* and GC content *g*, we regress the variance for the targets against both (i) the GC content of the targets and (ii) the mean read count across the reference samples for the targets with a generalized additive model, using the *mgcv* package in R (18).

### Hidden Markov model

To detect CNVs, we use a hidden Markov model (HMM) and the Viterbi algorithm to segment the targets into deletion, normal and duplication regions. To calculate the emission probabilities, we use the alternative parameterization of the negative binomial distribution with the mean and the size parameter *z*, where *μ_ij_* and $\sigma _{ij}^2$$\sigma _{ij}^2$ are related to the size parameter *z_ij_* via the relationship $z_{ij} = \mu _{ij}^2 /(\sigma _{ij}^2 - \mu _{ij} )$$z_{ij} = \mu _{ij}^2 /(\sigma _{ij}^2 - \mu _{ij} )$. To calculate the mean of the negative binomial distribution for the deletion and duplication states, we multiply *μ_ij_* by 1/2 and 3/2, respectively, to model a single-copy deletion and duplication. To calculate the size parameter for the deletion and duplication states, we likewise multiply *z_ij_* by 1/2 and 3/2, as the size parameter is roughly linearly related to the mean read count (Supplementary Figure S3).

The transition parameters used take into account the average rate of CNV occurrence in the exome, *p*, the average number of targets in a CNV, *T* and *D*, the expected distance between targets in a CNV, so that the farther apart two targets are the less likely they are to share the same state (10,16). To facilitate comparison with the XHMM algorithm, we use the XHMM default parameters: $p = 10^{ - 8}$$p = 10^{ - 8}$, *T* = 6 and *D* = 70 000 bases (10).

After detecting CNVs in the samples, we genotype each call in every sample as described in (10) by calculating a NQ and SQ score, allowing us to determine whether a CNV has likely been transmitted from a parent to a child, or whether a CNV in a child is *de novo*. For a given deletion or duplication called in sample *j* in a particular region, the ‘NQ’ score for that region for a sample *k* gives the Phred-scaled probability that sample *k* has no deletion or duplication, respectively, in that region. The ‘SQ’ score gives the Phred-scaled probability that any of the targets in that region have a deletion or duplication, respectively. By considering only CNV calls with ‘SQ’ score above a certain threshold, we can restrict our attention to a higher-quality call set than all the CNV calls (10).

After detecting CNVs, we calculate the most likely copy number for each CNV call by comparing the likelihood of a set of candidate copy number states, given the estimates of the parameters of the negative binomial distribution calculated above for each target in the segment. We consider the possibilities that there are 0, 1, 3, 4, 5 or 6 copies of the segment. Given the rarity of homozygous deletions and multiple copy duplications (i.e. 4, 5 and 6 copies), we require that the likelihood ratio for such events, compared to single copy events, be greater than a user-adjustable threshold, which we set equal to 1000. To calculate the mean and size parameter for the 4, 5 and 6 copy states, we multiply *μ_ij_* and *z_ij_* by 4/2, 5/2 and 6/2, respectively. To estimate the appropriate parameters for the 0 copy number state, i.e. homozygous deletion, we examined data in female samples for targets on the Y chromosome, where we expect to see read counts of 0. We observed that the counts observed for these targets in the female samples approximately followed a Poisson distribution with mean <1. In our data, the observed mean was ∼0.2, so we used a Poisson distribution with this mean to calculate the likelihood of a homozygous deletion (see Supplementary Note 1 for a further discussion of this parameter, which we expect will be platform-specific).

### Filtering

After calling CNVs, we find that those samples that have fewer samples with which their read count is highly correlated have a high number of CNV calls (Supplementary Figure S4). An examination of the relationship in these seven samples between the read count of each target and its GC content reveals that some have GC content–read count relationships that are markedly different from those of the remaining samples (Supplementary Figure S2), suggesting the influence of experimental conditions unique to these samples (and not the influence of unusual CNV profiles of these samples). Supplementary Figure S5 shows the average depth of coverage in each cluster. We apply a filter that excludes all calls for any sample that has more than *N*_max_ calls. Here, we set *N*_max_ = 50. In the dataset described in ‘Results’ section, this filter results in excluding calls for seven samples.

### Data processing

Binary Sequence Alignment/Map (BAM) files were generated using the ‘best practice’ bwa/GATK/Picard pipeline recommended by GATK. The mean sequence depth of the BAMs across the NimbleGen probes was 95.90, with standard deviation 19.19. Read counts for CANOES were generated using the getBamCounts function from the *ExomeDepth* packagein R, which is based on the countBamInGRanges function from the *ExomeCopy* package in R. To generate the XHMM results described below, the instructions in the XHMM documentation were followed and the default parameters were used, except that the discoverSomeQualThresh parameter was set to 0 so that XHMM would output all of its CNV calls, not just those whose quality score exceeds 30.

## RESULTS

We applied CANOES and XHMM to 328 samples, including 104 complete trios in which the probands had congenital heart disease. The samples were whole-exome sequenced at the Yale Center for Genome Analysis. Genomic DNA was captured using the NimbleGen v2.0 exome capture reagent (Roche) and sequenced (Illumina HiSeq, 75 base, paired-end reads), as described in (19). Because the analyses below rely on the presence of complete trios, the 16 samples not in complete trios have been excluded. Also, three trios have been excluded due to potential sample mix-up issues or lack of paternity. Finally, the six trios with members filtered out by CANOES due to too many CNV calls have also been excluded. Therefore, the analyses below are based on the results of 285 samples in 95 trios. Running CANOES on a 2.3 GHz CPU core took ∼6 min per sample.

### Number of CNV calls

Running CANOES on the autosomal chromosomes of 285 samples resulted in 2990 calls (2103 deletion calls and 887 duplication calls) after filtering. (Note that due to complications in calling CNVs on the sex chromosomes resulting from the samples being of different sexes, all analyses here are restricted to the autosomes.) Running XHMM on the same samples resulted in 3470 calls (1523 deletion calls and 1947 duplication calls). We therefore see that CANOES makes significantly more deletion calls than XHMM and significantly fewer duplication calls. Supplementary Figure S6 shows the distribution of the number of calls per sample for the two methods.

### Sensitivity

To measure the sensitivity of CANOES to rare CNVs, we first counted how many of the CANOES calls overlapped with rare (frequency <1%, as calculated by PLINK) (20) high-quality (>100 kb) calls made on the same samples by PennCNV (using Illumina 1M and 2.5M SNP chip data) that overlapped with one or more NimbleGen targets.

There were 174 low-frequency high-quality PennCNV calls in 118 of these 285 samples. CANOES and XHMM both detected 74% of these. Of these 174 PennCNV calls, 44 were deletions (in 37 samples). CANOES detected 73% of these, versus 66% for XHMM (see Tables 1a and b for separate sensitivities to PennCNV deletions and duplications overlapping different numbers of targets). Of the 130 low-frequency high-quality PennCNV duplication calls, CANOES detected 75%, versus 77% for XHMM. Overall, CANOES had higher sensitivity for deletions than XHMM, while making significantly more deletion calls, and slightly lower sensitivity for duplications, while making significantly fewer duplication calls. The PennCNV comparison suggests that CANOES and XHMM have comparable sensitivity, and that CANOES may have higher sensitivity for small deletions. Of the 13 high-quality PennCNV deletion calls that overlapped with three or fewer exome targets, CANOES detected six, versus four for XHMM. Of the 30 high-quality PennCNV duplication calls that overlapped with three or fewer exome targets, CANOES detected 12, versus 13 for XHMM.

**Table 1.** Sensitivity of CANOES and XHMM to PennCNV calls

Number of exome targets	Number of PennCNV calls	CANOES sensitivity	XHMM sensitivity
(a) *Deletions*: this table shows the proportion of high-quality PennCNV deletion calls overlapping from 1 to 10 exome targets that were detected by CANOES and XHMM			
1	44	32 (73%)	29 (66%)
2	37	28 (76%)	29 (78%)
3	34	28 (82%)	27 (79%)
4	31	26 (84%)	25 (81%)
5	26	22 (85%)	21 (81%)
6	25	22 (88%)	21 (84%)
7	22	19 (86%)	18 (82%)
8	22	19 (86%)	18 (82%)
9	20	18 (90%)	17 (85%)
10	18	16 (89%)	16 (89%)

(b) *Duplications*: this table shows the proportion of high-quality PennCNV duplication calls overlapping from 1 to 10 exome targets that were detected by CANOES and XHMM			
1	130	97 (75%)	100 (77%)
2	111	92 (83%)	94 (85%)
3	106	88 (83%)	90 (85%)
4	100	85 (85%)	87 (87%)
5	92	79 (86%)	81 (88%)
6	87	75 (86%)	77 (89%)
7	81	69 (85%)	71 (88%)
8	76	64 (84%)	66 (87%)
9	74	62 (84%)	64 (86%)
10	69	58 (84%)	60 (87%)

Higher sensitivity of CANOES for small deletions is apparent if instead of considering PennCNV calls >100 kb we consider the 946 PennCNV calls overlapping with 10 or more array probes, 776 (82%) of which are <100 kb in length (their median length is 22 kb). Of the 432 such PennCNV deletion calls, CANOES detected 128 (30%), whereas XHMM detected 106 (25%); of the 514 such PennCNV duplication calls, CANOES detected 242 (47%), versus 243 (47%) for XHMM (see Supplementary Table S2).

### Specificity

One way to measure the specificity of CANOES is to use data from a small number of experiments that were undertaken to try to validate some CNV calls made from both microarray and exome sequencing data from these samples. In all, 28 *de novo* CNV calls in these samples made by various methods, including XHMM and PennCNV, were subjected to experimental validation using digital droplet polymerase chain reaction (PCR) (21). Nineteen of these validation experiments resulted in confirmation. Of the 19 validated CNVs, 11 were called by both XHMM and CANOES, and the remaining eight were called by neither XHMM nor CANOES. One of the CNV calls that was detected by XHMM was not validated; this call was not made by CANOES.

Another way to measure the specificity of CANOES is to examine how many of the rare (frequency < 1%) CANOES calls coincided with PennCNV calls. There were 1324 such rare CANOES calls, and 112 (8%) coincided with PennCNV calls >100 kb in length. For purposes of comparison, there were 1805 such rare XHMM calls, and 112 (6%) coincided with PennCNV calls >100 kb in length. 107 of the 112 calls made by each of CANOES and XHMM were made jointly; each method made five PennCNV consistent calls that the other method did not. Three hundred and thirteen of the 1324 rare CANOES calls (24%) coincided with PennCNV calls overlapping with 10 or more probes (of which there were 946), as did 293 of the 1805 rare XHMM calls (16%).

To more thoroughly investigate the specificity of CANOES, we filtered the CANOES calls based on their quality scores and considered, at varying quality score thresholds, the median transmission ratio from parents to children. We can expect that the specificity of CANOES will be satisfactory if the median transmission ratio of the CANOES call set converges to 50% as the quality score threshold is raised. To determine the median transmission ratio for a given quality score threshold, for each parent-proband pair, we considered each deletion or duplication called in the parent with a quality score at or above that threshold. If the SQ score of the proband for a deletion or duplication, as applicable, exceeded the applicable NQ score of the proband, we considered the CNV to have been transmitted; otherwise, we considered the CNV not to have been transmitted. (We excluded as uncertain the very few CNVs for which the ‘SQ’ score equaled the ‘NQ’ score.) The transmission ratio was then calculated as the ratio between the number of transmitted CNVs and the total number of transmitted and not transmitted CNVs. Supplementary Figure S7 demonstrates that both XHMM and CANOES converge to a median transmission ratio of 50% as the quality is raised, albeit at different quality scores, CANOES at 90 and XHMM at 57. At these quality thresholds, CANOES made 1336 calls and XHMM made 1524. One hundred four (8%) of these CANOES calls overlapped with PennCNV calls >100 kb, and 105 (7%) of these XHMM calls overlapped with PennCNV calls >100 kb. Moreover, 941 of the calls were jointly made by CANOES and XHMM (see also Supplementary Figure S8).

**Figure 3.**
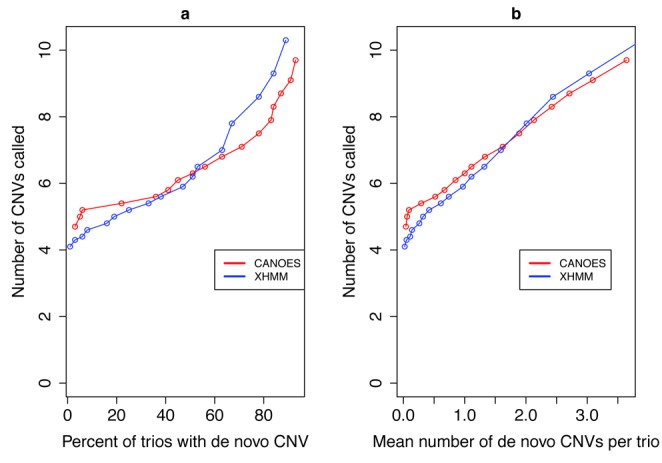
Number of CNVs called versus *de novo* CNVs. This plot shows, for each of CANOES and XHMM, the relationship between the number of CNVs called and the (**a**) percentage of trios with a *de novo* CNV and (**b**) mean number of *de novo* CNVs per trio. Moving along each curve from right to left, the quality score increases, and so the number of CNVs called as well as the (a) percentage of trios with a *de novo* CNV and (b) mean number of *de novo* CNVs per trio decreases. In the plausible range for the percentage of trios with a *de novo* CNV, CANOES makes more calls per sample than does XHMM.

Another measure of the specificity of CANOES is that, as the quality score is raised, the number of *de novo* CNVs called in the probands should fall, and only a small fraction of trios should have 1 or more *de novo* CNVs. Following XHMM, we consider a CNV in a child to be *de novo*, at a certain quality score threshold, if its ‘SQ’ quality exceeds the threshold and the ‘NQ’ quality in each of its parents also exceeds that threshold. Supplementary Table S3 shows that, in both CANOES and XHMM, the mean number of *de novo* CNVs per trio falls to 0 as the quality score is raised, as does the proportion of trios with at least one *de novo* CNV (see also Figure 3). In summary, for both XHMM and CANOES, using quality scores is a satisfactory way to improve the specificity of the call set of each method, and can result in a call set with good Mendelian properties.

### Effects of CNV size

**Figure 4.**
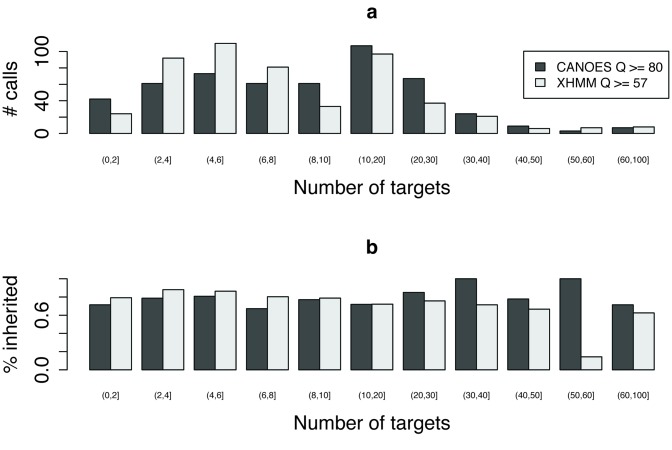
Comparison of calls of different size. (**a**) Number of calls in probands as a function of number of targets, including XHMM calls with quality scores ≥57 and CANOES calls with quality scores ≥80. The CANOES quality threshold of ≥80 was selected because the total number of calls for CANOES at this threshold was nearly the same as the corresponding number of XHMM calls with quality ≥57. The notation (*a*,*b*] indicates that the bars above include samples with *a* + 1 through *b* calls. (**b**) Percentage of calls in probands that appear to be inherited (SQ score > NQ score, in either parent) for CANOES and XHMM, as a function of the number of targets. XHMM made seven calls 51–60 targets long. One overlapped with a call made by CANOES and PennCNV and showed evidence of having been inherited, but the other six did not. Notably, these six calls were in two genomic locations, one in chromosome 14 (three calls) and one in chromosome 19 (three calls). XHMM made three additional long calls in these regions in the parents. The absence of overlap with CANOES and PennCNV and the many calls in these regions in parents and probands suggests that these are artifacts, not *de novo* CNVs.

To investigate the properties of CANOES and XHMM with respect to CNVs of different sizes, we binned the CNV calls made by each method according to how many targets they overlapped with, and calculated, for each bin, the percentage of CNVs called in probands that appeared to be inherited from either mother or father. We counted a CNV as ‘inherited’ if the SQ score of either parent for a deletion or duplication in the region, as applicable, exceeded the applicable NQ score of the either parent. We expect that the number of *de novo* CNVs should be low and so we can use the fraction of CNVs in probands that are inherited as a proxy for the accuracy of these calls. Figure 4a illustrates that CANOES made more short CNV calls (overlapping 1 or 2 targets) and more long calls (>8 targets) than XHMM. Supplementary Figure S10 shows that, for overlapping XHMM–CANOES calls, the CANOES call tends to be longer than the corresponding XHMM call. The fraction inherited is generally comparable between CANOES and XHMM (Figure 4b). We note that among the short XHMM calls that overlapped with CANOES calls, 12 out of 13 (92%) appear to be inherited.

**Figure 5.**
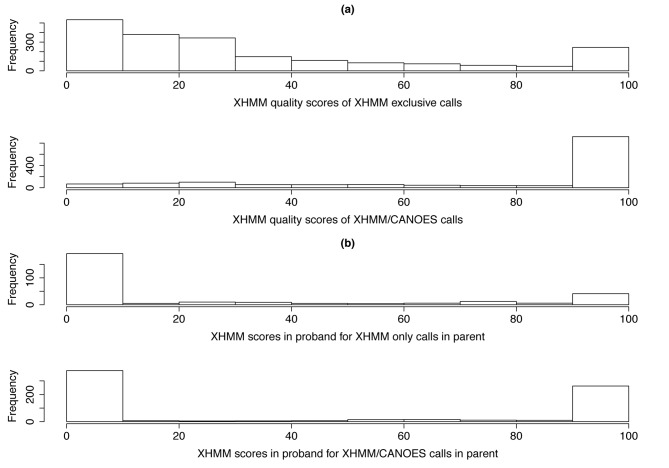
Overlap between XHMM and CANOES calls. (**a**) Histogram of quality scores of CNV calls. These histograms show the distribution of XHMM quality scores for those calls made by XHMM alone (top) and those calls made by XHMM that overlapped with CANOES calls (bottom). (**b**) XHMM Quality score in probands of high-quality CNV calls made in parents. The top histogram shows, for XHMM calls with high quality scores (≥57) in parents made only by XHMM, the strength of the evidence (the XHMM quality score in the child) that the parent CNV was transmitted to the child. The bottom histogram shows the same for XHMM calls that overlapped with CANOES calls.

### Overlap between XHMM and CANOES

To investigate whether the calls jointly made by XHMM and CANOES were of especially high accuracy and thus could be used for filtering purposes, we compared the quality of the calls made by XHMM alone to the calls made jointly by XHMM and CANOES. The histograms in Figure 5(a) show that a much higher proportion of the jointly made calls have high XHMM quality scores than do the calls made by XHMM alone.

To further compare the quality of these two categories of calls, we focused on the high-quality XHMM calls (quality scores ≥ 57) in parents. To see whether the quality of such calls made by XHMM alone was significantly different from the quality of such calls made jointly by XHMM and CANOES, we compared the transmission characteristics of each category of CNV calls. For each category, therefore, we examined the XHMM quality scores of those calls in the applicable probands, which we treat as a proxy for the probability that the call in the parent was a real call and not an artifact. The histograms in Figure 5(b) show that a much larger proportion of the overlapping calls have high XHMM quality scores and therefore appear to have been transmitted than the calls made by XHMM alone. This suggests that the jointly made calls are of significantly higher accuracy than the calls made by XHMM alone.

We also investigated the *de novo* rate of the high quality XHMM calls (quality scores ≥ 57) in probands. In sum, XHMM made 521 such high-quality calls in probands (Table 2). Three hundred and fifty-five of these calls coincided with CANOES calls and 166 did not. Of the calls that coincided with CANOES calls, 10 (2.8%) were *de novo*, determined using XHMM quality scores. Of the calls made by XHMM alone, 39 (23.5%) were *de novo*. Therefore, the calls that overlap with CANOES have a much lower and potentially more realistic *de novo* rate (22), considering general limits on the power of detecting CNVs from exome data (9). For purposes of comparison, we investigated the *de novo* rate of high quality CANOES calls (quality score ≥ 80) in probands. We selected this quality score since it provided a call set in probands of similar size to the XHMM ≥ 57 call set. Of the 187 calls made by CANOES alone, 3 (1.6%) were *de novo*, determined using CANOES quality scores.

**Table 2.** Rate of *de novo* CNVs in XHMM/CANOES overlapping calls and XHMM and CANOES exclusive calls

	CANOES exclusive	XHMM/CANOES overlap	XHMM exclusive
Total number of calls in probands	187	355	166
*de novo* (percentage of total)	3 (1.6%)	10 (2.8%)	39 (23.5%)

This table compares the number of total and high confidence *de novo* CNV calls in the XHMM (quality ≥ 57)/CANOES (quality ≥ 0) overlapping set, the XHMM (quality ≥ 57) exclusive set and the CANOES (quality ≥ 80) exclusive set. The CANOES quality threshold of 80 was selected because at this quality threshold CANOES makes nearly the same number of calls in probands as does XHMM at 57. A call was considered to be *de novo* in a child at a certain threshold if its ‘SQ’ quality exceeded the threshold and the ‘NQ’ quality in each parent also exceeded the threshold.

### Using XHMM and CANOES with fewer reference samples

To compare the performance of XHMM and CANOES using fewer reference samples, we picked at random five trios from among the samples in the largest cluster of samples, and ran XHMM and CANOES on this smaller dataset. We then compared the results from each method with the results from that method obtained using all the available reference samples, using the latter in each case as a pseudo ‘gold standard’. Table 3 shows that the results from CANOES using few reference samples were significantly more concordant with the results using all reference samples than were the XHMM results.

**Table 3.** Comparison of results using 15 and 328 reference samples for CANOES and XHMM

	CANOES	XHMM
Number of calls when using 328 reference samples	135	138
Number of calls when using 15 reference samples	196	206
Number of overlapping calls when using 15 and 328 reference samples	118	90
Recall	118/135 (87%)	90/138 (65%)
Precision	118/196 (60%)	90/206 (44%)

This table compares CANOES results when using 15 reference samples to CANOES results using 328 samples, and likewise for XHMM. One sample filtered out by CANOES was excluded from the results below.

## DISCUSSION

We have presented a computational method (CANOES) to detect rare CNVs in exome sequencing studies that has high sensitivity for small events. We also demonstrated that it can effectively be used in conjunction with XHMM to filter for high-quality CNV calls. CANOES has high specificity, as demonstrated by the Mendelian properties of the CANOES call set when its quality threshold is raised, and high sensitivity to high-quality microarray-based CNV calls. With respect to small deletions, CANOES may have superior sensitivity to XHMM.

As suggested by its higher sensitivity for small deletions, CANOES may provide calling superior to XHMM for samples with large numbers of well-matched reference samples, especially for small events. CANOES may also provide superior calling where the average depth of coverage is lower, or where fewer reference samples are available. CANOES would also be suitable for high-depth targeted sequencing projects. CANOES is, however, limited by the correlation structure of the read count matrix of the available exome samples. For those samples subject to unique experimental conditions, reliable results will not be achievable, whereas XHMM can still make high-quality CNV calls in some of these samples. A multi-dimensional scaling plot of the correlation matrix is an effective tool for determining which (and how many) samples will likely not be susceptible to CNV calling with CANOES. As demonstrated by the superior performance of XHMM on these samples, XHMM will be a better choice for samples that are poorly correlated with the bulk of the available reference samples. We have further shown that CANOES and XHMM can be used together to efficiently screen CNV call sets for high quality calls, and that using CANOES and XHMM together may lead to a higher quality call set than using either method alone. This will facilitate selection of high-quality candidate CNVs for experimental validation and statistical association.

CANOES may offer superior performance to XHMM in studies where few samples are available, as the performance of PCA-based methods is worse when the number of reference samples is <50, whereas CANOES can use fewer reference samples (as demonstrated here, as low as 15). CANOES should work with capture platforms besides NimbleGen v2.0, although all reference samples used in one run of CANOES should have been processed with the same platform.

One possible extension to the model presented here is to substitute the point estimate with a posterior distribution for the variance of read count at each exome target. Explicitly taking into account the uncertainty in the determination of this variance might lead to more accurate quality scores, and therefore better filtering of the CANOES call set. Another possible extension is to correct the read counts for the GC content of the targets, which may make it possible to call CNVs effectively in some of the samples that CANOES currently filters out. Finally, a more robust model of read count in areas of homozygous deletion would lead to more accurate genotyping of this important class of events.

CANOES has advantages in detecting small CNVs by modeling read counts using a more precise distribution (negative binomial) than Gaussian approximation. Moreover, CANOES is complementary to XHMM and similar PCA-based methods, and by combining CANOES and XHMM results, one can produce highly accurate calls that are not achievable by one method only. It may greatly enhance the utility of exome sequencing in disease studies.

An R package incorporating the functionality of CANOES is available for download. The R package, documentation and source code may be obtained from http://www.columbia.edu/∼ys2411/canoes/.

## SUPPLEMENTARY DATA

Supplementary data are available at NAR Online, including [1].

SUPPLEMENTARY DATA
