# Supplementary material for: CANOES: detecting rare copy number variants from whole exome sequencing data
Source: Nucleic Acids Res. 2014 Apr 25;42(12):e97. doi: 10.1093/nar/gku345 (PMC4081054; doi:10.1093/nar/gku345)
Supplement: SUPPLEMENTARY DATA [file supp_42_12_e97__index.html]

CANOES: detecting rare copy number variants from whole exome sequencing data — CANOES: detecting rare copy number variants from whole exome sequencing data — SUPPLEMENTARY DATA 

# CANOES: detecting rare copy number variants from whole exome sequencing data

## SUPPLEMENTARY DATA

**Files in this Data Supplement:**

- SUPPLEMENTARY DATA
