# Supplementary material for: CANOES: detecting rare copy number variants from whole exome sequencing data
Source: Nucleic Acids Res. 2014 Apr 25;42(12):e97. doi: 10.1093/nar/gku345 (PMC4081054; doi:10.1093/nar/gku345)
Supplement: SUPPLEMENTARY DATA [file supp_gku345_nar-03528-z-2013-File007.docx]

# Supplementary figures, tables and notes

# **CANOES: Detecting rare copy number variants from whole exome sequencing data**

Daniel Backenroth, Jason Homsy, Laura R. Murillo, Joe Glessner, Edwin Lin, Martina Brueckner, Richard Lifton, Elizabeth Goldmuntz, Wendy K. Chung, Yufeng Shen

### Supplementary Figure 1 - Conceptual overview of algorithm

The figure shows a conceptual overview of the computational steps taken by CANOES, starting with BAMs and ending with a segmentation of the exome targets into normal, deletion and duplication regions using a hidden Markov Model and assignment of most likely copy numbers using maximum likelihood.

### Supplementary Figure 2 - Outlier samples

This plot shows loess curves for the readcount-GC relationship among all targets of median width for each of the samples in the dataset described in (Section *Results*). The curves for the 7 samples filtered out by CANOES are colored black. The colors of the other samples correspond to the colors used in Figure 1, showing that the MDS plot in Figure 1 separates groups of samples with different readcount-GC relationships.

###

### Supplementary Figure 3 - Relationship of size parameter to mean read count

### This plot shows that the observed size parameter of the read count at a target across reference samples increases approximately linearly with the mean read count at the target.

###

### Supplementary Figure 4 - Number of calls and outlying samples

This plot, like Figure 1, shows a 2-dimensional scaling of the inverse of the covariance matrix of the read count data, except that the samples with more than 50 CNV calls are colored in black. The sample excluded from Figure 1 is included in this plot (at middle left).

###

### Supplementary Figure 5 - Relationship of clustering to depth

These plots show the distribution of average sequencing depth for the four groups of samples colored in distinct colors in Figure 1. The samples in the red and green clusters have a similar distribution of average sequencing depth, while the samples in the blue cluster have lower average sequencing depth. There are only two purple samples.

### Supplementary Figure 6 - Number of CNV calls per sample

This figure shows the distribution of the number of CNV calls per sample made by CANOES and XHMM for the data set described in (Section *Results*). The notation (a,b] indicates that the bars above include samples with a+1 through b calls.

### Supplementary Figure 7 – Transmission ratio

These boxplots show the parent-to-child transmission rates of CNV calls made by CANOES (a) and XHMM (b) in the parents as a function of each method's quality score.

###

### Supplementary Figure 8 – Recall vs. precision using PennCNV calls as “gold standard”

These plots shows how the recall (using as a "gold standard" the percent of rare (frequency < 1%) PennCNV calls that are recovered by CANOES or XHMM, as applicable) varies with 1 minus the precision (percent of rare (frequency < 1%) calls by CANOES or XHMM, as applicable, that overlap with a PennCNV call, again using PennCNV as a "gold standard") for CANOES and XHMM as the quality score threshold for each method is varied from 90 (lower left hand corner) to 0 (upper right hand corner).

Figure (a) includes PennCNV calls greater than 100 kb in length, and figures (b), (c) and (d) include PennCNV calls overlapping at least 5, 10 and 20 probes, respectively. See Figure 3 for this plot including PennCNV calls overlapping at least 10 probes.

### Supplementary Figure 9 – Number of CNVs called vs. *de novo* CNVs

This plot shows, for each of CANOES and XHMM, the relationship between the number of CNVs called and the (a) percent of trios with a *de novo* CNV and (b) mean number of *de novo* CNVs per trio. Moving along each curve from right to left, the quality score increases, and so the number of CNVs called as well as the (a) percent of trios with a *de novo* CNV and (b) mean number of *de novo* CNVs per trio decreases. In the plausible range for the percentage of trios with a *de novo* CNV, CANOES makes more calls per sample than does XHMM.

### Supplementary Figure 10 – Lengths of CNV calls for overlapping calls

This histogram shows, for the overlapping calls between CANOES and XHMM, the difference in length between the CANOES call and the corresponding XHMM call. CANOES calls tend to be longer than XHMM calls.

### Supplementary Table 1 - Power analysis

Using samples from one of the clusters shown in Figure 1, we calculated the variance of read count at targets as a function of read count. Different targets, depending on their GC content and other factors, including the presence of common CNVs, have more or less variable read count. For targets whose variability was at the 50th percentile, we calculated the theoretical power for detecting a 1 target deletion or duplication when the read count for the target is 110, 210, 310 and 410, using both the negative binomial (NB) and normal distributions. These correspond roughly to the median read counts when depth is 70X, 130X, 190X and 260X. These power calculations were done assuming a Type I error of 10^-5^.

| Read count for target | Power for detecting one target deletion using NB | Power for detecting one target duplication using NB | Power for detecting one target deletion using normal distribution | Power for detecting one target duplication using normal distribution |
| --- | --- | --- | --- | --- |
| 110 | 19% | 3% | 0% | 7% |
| 210 | 72% | 24% | 30% | 37% |
| 310 | 87% | 39% | 59% | 53% |
| 410 | 92% | 48% | 73% | 63% |

### Supplementary Table 2 - Sensitivity of CANOES and XHMM to PennCNV calls overlapping with 10 or more array probes

(2a) *Deletions:* this table shows the proportion of PennCNV deletion calls overlapping with 10 or more array probes that were detected by CANOES and XHMM.

| Number of exome targets | Number of PennCNV calls | CANOES sensitivity | XHMM sensitivity |
| --- | --- | --- | --- |
| 1 | 432 | 128 (30%) | 106 (25%) |
| 2 | 247 | 110 (45%) | 101 (41%) |
| 3 | 207 | 98 (47%) | 89 (43%) |
| 4 | 177 | 81 (46%) | 76 (43%) |
| 5 | 150 | 66 (44%) | 62 (41%) |
| 6 | 133 | 61 (46%) | 57 (43%) |
| 7 | 112 | 48 (43%) | 44 (39%) |
| 8 | 100 | 43 (43%) | 40 (40%) |
| 9 | 83 | 34 (41%) | 33 (40%) |
| 10 | 74 | 28 (38%) | 28 (38%) |

(2b) *Duplications:* this table shows the proportion of PennCNV duplication calls overlapping with 10 or more array probes that were detected by CANOES and XHMM.

| Number of exome targets | Number of PennCNV calls | CANOES sensitivity | XHMM sensitivity |
| --- | --- | --- | --- |
| 1 | 514 | 242 (47%) | 243 (47%) |
| 2 | 365 | 225 (62%) | 229 (63%) |
| 3 | 328 | 206 (63%) | 211 (64%) |
| 4 | 282 | 186 (66%) | 191 (68%) |
| 5 | 234 | 156 (67%) | 161 (69%) |
| 6 | 206 | 140 (68%) | 144 (70%) |
| 7 | 177 | 122 (69%) | 125 (71%) |
| 8 | 162 | 112 (69%) | 115 (71%) |
| 9 | 144 | 101 (70%) | 104 (72%) |
| 10 | 132 | 95 (72%) | 98 (74%) |

### Supplementary Table 3 - Characteristics of CNV call set at different quality thresholds

This table shows, at different quality thresholds, the mean and median number of CNV calls per sample, the mean and median number of *de novo* CNV calls per trio, and the percent of trios with at least one *de novo* CNV call for CANOES and XHMM.

|  | | CANOES sensitivity | | | | | | XHMM sensitivity | | | | |
| --- | --- | --- | --- | --- | --- | --- | --- | --- | --- | --- | --- | --- |
| Quality threshold | | mean (median) number of calls | | mean (median) number of *de novo* calls | | percent of trios with *de novo* calls (%) | | mean (median) number of calls | | mean (median) number of *de novo* calls | | percent of trios with *de novo* calls (%) |
| 10 | 9.7 (9) | | 3.64 (3) | | 93 | | 10.3 (10) | | 3.89 (3) | | 89 | |
| 15 | 9.1 (9) | | 3.09 (2) | | 91 | | 9.3 (9) | | 3.03 (2) | | 84 | |
| 20 | 8.7 (8) | | 2.71 (2) | | 87 | | 8.6 (8) | | 2.44 (2) | | 78 | |
| 25 | 8.3 (8) | | 2.42 (2) | | 84 | | 7.8 (7) | | 2.01 (1) | | 67 | |
| 30 | 7.9 (7) | | 2.13 (1) | | 83 | | 7 (7) | | 1.59 (1) | | 63 | |
| 35 | 7.5 (7) | | 1.89 (1) | | 78 | | 6.5 (6) | | 1.32 (1) | | 53 | |
| 40 | 7.1 (7) | | 1.62 (1) | | 71 | | 6.2 (6) | | 1.11 (1) | | 51 | |
| 45 | 6.8 (6) | | 1.33 (1) | | 63 | | 5.9 (6) | | 0.97 (0) | | 47 | |
| 50 | 6.5 (6) | | 1.11 (1) | | 56 | | 5.6 (5) | | 0.74 (0) | | 38 | |
| 55 | 6.3 (6) | | 1 (1) | | 51 | | 5.4 (5) | | 0.61 (0) | | 33 | |
| 60 | 6.1 (6) | | 0.85 (0) | | 45 | | 5.2 (5) | | 0.42 (0) | | 25 | |
| 65 | 5.8 (6) | | 0.67 (0) | | 41 | | 5 (5) | | 0.32 (0) | | 19 | |
| 70 | 5.6 (5) | | 0.52 (0) | | 36 | | 4.8 (5) | | 0.26 (0) | | 16 | |
| 75 | 5.4 (5) | | 0.29 (0) | | 22 | | 4.6 (4) | | 0.14 (0) | | 8 | |
| 80 | 5.2 (5) | | 0.09 (0) | | 6 | | 4.4 (4) | | 0.11 (0) | | 6 | |
| 85 | 5 (5) | | 0.06 (0) | | 5 | | 4.3 (4) | | 0.05 (0) | | 3 | |
| 90 | 4.7 (4) | | 0.04 (0) | | 3 | | 4.1 (4) | | 0.02 (0) | | 1 | |

### Supplementary Note 1 - Homozygous deletions

We examined another data set, in which a different capture platform was used (Illumina TruSeq) and observed a distribution of read counts in females on the Y chromosome (excluding pseudoautosomal regions and known X chromosome-homologous regions ([1](#_ENREF_1))) similar to that described in the main text. The parameter for the Poisson mean of expected read count for homozygously deleted targets is user-adjustable, and users may wish to examine their data before setting this parameter. We recommend that users exclude targets with extreme variability in read count prior to calculating the mean and variance of the read count of targets on the Y chromosome in females, to exclude the effect of targets with high sequence homology to targets on other chromosomes. We expect that after excluding such targets that the distribution of read counts will be approximately Poisson, with a very low mean.

The estimation of read count in areas of homozygous deletion is difficult given the rarity of such events, and may not be well approximated by the distribution of read count on the Y chromosome in females. Users should therefore be cautious in interpreting the most likely copy number state as estimated by CANOES.

1. Wilson, N.D., Ross, L.J.N., Close, J., Mott, R., Crow, T.J. and Volpi, E.V. (2007) Replication profile of PCDH11X and PCDH11Y, a gene pair located in the non-pseudoautosomal homologous region Xq21.3/Yp11.2. *Chromosome Res*, **15**, 485-498.
